# Medications Adherence and Associated Factors among Patients with Type 2 Diabetes Mellitus in the Gaza Strip, Palestine

**DOI:** 10.3389/fendo.2017.00100

**Published:** 2017-06-09

**Authors:** Aymen Elsous, Mahmoud Radwan, Hasnaa Al-Sharif, Ayman Abu Mustafa

**Affiliations:** ^1^Department of Health Management and Economics, School of Public Health, Tehran University of Medical Sciences – International campus, Tehran, Iran; ^2^Quality Improvement and Infection Control Office, Shifa Medical Complex, Gaza Strip, Palestine; ^3^Directorate General of International Cooperation, Ministry of Health, Gaza Strip, Palestine; ^4^Director of Chronic Diseases Department, Al Rimal Martyrs Health Center, Ministry of Health, Gaza Strip, Palestine; ^5^Department of Research, Directorate General of Human Resources Development, Ministry of Health, Gaza Strip, Palestine

**Keywords:** medication adherence, diabetes mellitus, factors, Palestine, cross-sectional

## Abstract

**Aim:**

The aim of this study was to evaluate the adherence to anti-diabetic medications among patients with type 2 diabetes mellitus (DM) seeking medical care in the Gaza Strip, Palestine.

**Methods:**

A cross-sectional study was conducted among 369 primary care patients with type 2 DM from October to December 2016. Adherence to medications was measured using the Morisky Medication Adherence Scale (MMAS-4). Socio-demographic and clinical variables, provider–patient relationship, health literacy, and health belief were examined for each patient. Univariate, binary logistic regression and multiple linear regression were applied to determine the independent factors influencing adherence to anti-diabetic medications using SPSS version 22.

**Results:**

Of all the respondents, 214 (58%), 146 (39.5%), and nine (2.5%) had high (MMAS score = 0), medium (MMAS score = 1 + 2), and low (MMAS score ≥ 3) adherence to anti-diabetic medications, respectively. Factors that were independently associated with adherence to anti-diabetic medications were as follows: female gender [odds ratio (OR): 1.657, 95% confidence interval (CI): 1.065–2.578] and perception of disease’s severity (OR: 1.510, 95% CI: 0.410–5.560). Elderly (*t* = 1.345) and longer duration of DM (*t* = 0.899) were also predictors of adherence but showed no statistical significance (*p* > 0.05).

**Conclusion:**

The level of complete adherence to anti-diabetic medications was sub-optimal. New strategies that aim to improve patients’ adherence to their therapies are necessary taking into consideration the influencing factors and the importance of having diabetes educators in the primary care centers.

## Introduction

Diabetes mellitus (DM) is a serious and a rapidly growing public health problem that affects millions of people. It usually co-exists with other medical conditions, and its prevalence is increasing year by year reaching epidemic proportions. Currently, 387 million people have DM (1) and are expected to reach 483 million and 592 million by the year 2030 (2) and 2035 (1), respectively, in which type 2 DM is accounting 90% of cases (3). DM is responsible for more than 77% of morbidities and 88% of deaths in developing countries (1, 4).

It is noteworthy mentioning that 15 years later the Arab world in North Africa, the Middle East including Gulf area, is going to have a tremendous increase in the percentage of people having DM compared to other parts of the world (5). According to the International Diabetes Federation, the prevalence of DM in the Middle East and North African region will be the highest compared to other parts of the world (6).

In Palestine, few studies were conducted and the prevalence of DM was higher in urban areas compared to rural areas (7–9). Abu-Rmeileh and colleagues (10) estimated the prevalence of DM in Palestine at 20.8 and 23.4% in 2020 and 2030, respectively. Reports of the Ministry of Health (MoH) revealed that 27,601 have DM in the Gaza Strip with an incidence rate of 15.4/1,000 (11) and is steadily increasing because Gazian people are in a prolonged state of stress from political uncertainty and frequent violation. DM is known to be the disease of elderly; however, it is now more prevalent among younger age below 55 years old. Patients with DM are in an unhealthy class due to lack of knowledge about the disease and prevention of complications. Moreover, the increasing rate of obesity is the major risk factor for DM (12). Complications from DM, including diabetic foot, were previously studied in Gaza and showed to be significantly associated with a low educational level, low income, and primary care physicians’ attitudes (13, 14). DM represents 11.2% of all deaths (11), and half of physicians and nurses do not adhere to Palestinian diabetes guideline because of lack of interest (15) making real challenges to the health care services and proper diabetes management. Diabetic neuropathy is one of the major complications that presents in 12% of type 2 DM patients and is the leading cause of extremity amputations (11).

Achieving glycemic control and preventing early complications are the ultimate targets of diabetes management which depends on patient’s adherence to regimens (16). Adherence to prescribed medications is one of the key dimensions of health care quality, which is defined as the proportion of prescribed doses of medication actually taken by a patient over a specified period of time (17). Adherence to or compliance with medications has significant economic and therapeutic consequences (18), because non-adherence patients are at greater risk of developing complications affecting their health status and overall quality of life (19).

Patients’ adherence to their anti-diabetic medications is a critical and important factor to prevent serious undesirable complications and to reduce the health care resource utilizations. Effective diabetes management mandates good provider–patient relationship, and compliance to therapies is one of the significant aspects of the relation. Poor adherence to therapies is common, especially when comorbidities exist (20), and is believed to be influenced by several factors divided into five categories: patient-centered factors, therapy-related factors, health care system factors, social and economic factors, and disease-related factors (21–23).

Studies on adherence to anti-diabetic medications among type 2 DM patients in Palestine are few (24–26), but unfortunately none of these were carried out in the Gaza Strip. It became necessary and urgently needed to highlight the current status of adherence to diabetic medications among type 2 DM patients in the area of Gaza Strip and show the cultural differences within the same Palestinian population, with Arab-speaking countries and the overseas world. Thus, the aim of this study was to evaluate the adherence to anti-diabetic medications and examine the associated factors among type 2 DM patients attending primary health care clinics in the Gaza Strip. In addition, this study was based on the following hypotheses:

– Adherence to anti-diabetic medication is sub-optimal among primary health care patients with type 2 DM in the Gaza Strip.
– Elderly, duration of DM, health literacy, perception of disease’s severity and treatment’s benefit, and male gender are associated with adherence to medications.

## Materials and Methods

### Study Design and Setting

To elaborate the study data, a cross-sectional analytical design was used in 2016. The study was conducted in the primary health centers (PHCs; level IV) distributed across four Gaza governorates. They were 9 and serve 6,486 type II diabetic patients. A cluster sample was used to select one PHC (level IV) from each governorate (North, Gaza, Middle Zone, and South) in the Gaza Strip. The four named selected PHC beginning from the north were Jabalia martyrs, Al-Rimal martyrs, Dier Al-Balah martyrs, and Khan Younis martyrs, respectively.

### Sample and Sampling

Selection criteria for patients’ inclusion were the following: type 2 DM, at least 30 years old, under anti-diabetic therapies for at least 6 months at the time of enrollment, and willingness to participate in the study. Pregnant women and patients with mental diseases were excluded. The following formula was used to calculate the sample size, *n* = [(Z a/2)2 p(1 p)/d2]. Giving the prevalence of DM in Palestine equals or close to 10% (7–9), the sample size was 338. Adding 10% of non-response rate, the sample size for this study was 372 patients with type 2 DM. Then, it was decided to sample proportionally to achieve representativeness of sample and gender. A list of attending patients was prepared, and randomization was applied to select the first patient; then, the next was every 17th patient (6,486/372). In case the patient refused to participate, we took the next one without affecting the listing order.

### Data Collection

Adherence to prescribed therapies is believed to be influenced by other elements beyond the traditional socio-demographic or clinical-related factors (27). We used a structured questionnaire that has five parts: socio-demographic (age, gender, place of living, duration of DM, complications, previous hospitalization, etc.), health belief model (HBM; 16 items), patient–physician relationship (10 items), health literacy (4 items), and adherence to anti-diabetic medications (four items). The questionnaire was translated into Arabic and validated following the translation steps given by the Agency for Health Care Research and Quality (28). Internal consistency of the entire questionnaire examined with Cronbach’s alpha value was 0.76. Content validity was examined, by nine health experts, through the determination of item content validity index (I-CVI) and scale content validity index (S-CVI). The I-CVI and S-CVI ranged between 0.88–1.00 and 0.97–1.00, respectively. Inter-rater reliability measured by kappa statistic revealed a high agreement between nine raters (*k* ≥ 0.89). Three trained interviewers collected the required data by face-to-face interview. Data collection took place during patient visit to the PHC, and 15 min were enough to complete the questionnaire.

#### Health Beliefs of Patients Regarding DM

We used the HBM which was originally formulated to explain why persons would or would not undertake preventive health actions (29) and later applied to the prediction of compliance with prescribed therapies (30). The model is derived from psychological and behavioral theories and was conceptualized to enclose four elements: perceived susceptibility, perceived severity, perceived benefits, and perceived barriers. The HBM incorporated a 5-point Likert scale (1 = strongly disagree to 5 = strongly agree). The entire Cronbach’s alpha was 0.70. A score of ≥3 out of 5 demonstrated that patient had health beliefs regarding DM, while negatively worded questions with the score of <3 demonstrated bad health belief.

#### Patient–Provider Relationship

Physician–patient relationship was examined by patient–physician relationship questionnaire developed by Van der Feltz-Cornelis and colleagues (31). The questionnaire has 10 items and is answered on 5-point Likert scale from 1 = strongly disagree to 5 = strongly agree. The Cronbach’s alpha value of the entire 10 questions was 0.79. A score of ≥3 out of 5 demonstrates a good relationship between physician and patient.

#### Health Literacy

The health literacy of patients was measured using the Short Health Literacy Screening Tool (Brief Health Literacy Screening Tool; BRIEF) (32). This scale has four questions on 5-point Likert scale (1 = never to 5 = always). Internal consistency was measured with Cronbach’s coefficient alpha which was 0.71. A score of ≥3 out of 5 for positively worded questions demonstrates good health literacy and the score of <3 is bad health literacy.

#### Adherence to Anti-Diabetic Medications

We used the four-items Morisky Medications Adherence Scale (MMAS-4) which demonstrated a good concurrent and predictive validity and good internal consistency (33). This scale is answered with “yes” or “no.” Because the four questions are negatively coded items, 0 point is given to “no” answer and 1 point is given to “yes” answer: the less score the more adherence. The score is from 0 to 4 and is classified as follows: 0 = high adherence, 1–2 = medium adherence, 3–4 = low adherence. Internal consistency was examined, and Cronbach’s coefficient alpha was 0.72.

### Statistical Analysis

The Statistical Package for Social Sciences (SPSS) version 22 (IBM Corp, Armonk, NY, USA) was used in data analysis, and the data were checked for outliers and errors during data entry phase. Descriptive statistics included percentages and frequencies were calculated for categorical variables, while mean and SD for continuous variables. Items’ responses were collapsed into three choices “agree and strongly agree, do not know, and disagree and strongly disagree” for the patient–provider relationship and HBM questionnaires, and “always and sometimes, do not know, and rare and never” for health literacy questionnaire. Univariate analysis of data was used to determine the level of adherence, summarize the data, and describe the socio-demographic characteristics of participated patients. Crude odds ratios [ORs; 95% confidence interval (CI)] were used to describe the strength of association between independent variables and the dependent variable (adherence to medications). Independent binary or multi-group variables, with *p*-value of 0.25 or less, were taken for binary logistic regression and multiple linear regression analysis, respectively, to determine which variables were independently associated with the dependent variable. The frequently used *p*-value of ≤0.05 usually fails to recognize significant variables (34).

## Results

Three hundred and sixty-nine patients participated in the study, and their mean age (±SD) was 56.38 ± 10.36. More than half (55.8%) were females and 352 (95.4%) were married. With regard to clinical characteristics, 180 (48.8%) have no comorbidities; 42 (11.4%) have hypertension; 26 (7%) have retinopathy, and 104 (28.2%) presented with nephropathy. A majority of patients (321; 87%) were not previously hospitalized and were under oral hypoglycemic agent (241; 65.3%), while 85 (23%) were under insulin treatment. Mean duration (±SD) of having DM was 10.48 (8.12) and two-third of them have DM for more than 5 years (234; 63.4%). Good glycemic control (≤6.4%) was found in 41 (11.1%) patients.

### Response by Items

Answers for patient–physician relationship, health literacy, and HBM were collapsed into three main choices, and the mean (SD) is presented accordingly (Table 1). Mean score (SD) of HBM and patient–provider relationship questionnaire were above the recommended cutoff which demonstrates good health belief about DM and relationship between patients and their providers [3.6 (0.38) and 4.4 (0.55), respectively]. However, mean score (SD) of health literacy questionnaire was below the recommended cutoff which means bad health literacy [2.9 (0.59)].

**Table 1:** **Mean (SD), percentage of agree, do not know, and disagree of questionnaire items**.

Questions	M (SD)	Agree, *n* (%)	Do not know, *n* (%)	Disagree, *n* (%)
A: Health belief model	3.6 (0.38)			
My diabetes is well controlled	3.0 (1.24)	161 (43.6)	9 (2.4)	199 (53.9)
My diabetes would be worse if I did nothing about it	4.5 (0.6)	361 (97.8)	2 (0.5)	6 (1.6)
I believe my medication will help prevent complications related to diabetes	4.5 (0.62)	358 (97)	5 (1.4)	6 (1.6)
Diabetes can be a serious disease if I do not control it	4.6 (0.69)	354 (95.9)	1 (0.3)	14 (3.8)
My diabetes is not a problem for me as long as I feel all right	3.6 (1.35)	236 (64)	4 (1.1)	129 (35)
^a^My diabetes will have bad effect on my future health	1.6 (0.65)	350 (94.9)	12 (3.3)	7 (1.9)
^a^My diabetes will cause me to be sick a lot	1.6 (0.66)	353 (95.7)	6 (1.6)	10 (2.7)
I believe I will always need my diabetic medications	4.4 (0.77)	349 (94.6)	2 (0.5)	18 (4.9)
I believe I can control my diabetes	3.6 (1.22)	248 (67.2)	3 (0.8)	118 (32)
I believe that my medications will control my diabetes	4.3 (0.74)	348 (94.3)	2 (0.5)	19 (5.1)
If I change my eating habits, it will probably help me	4.2 (0.94)	323 (87.5)	5 (1.4)	41 (11.1)
My medicine makes me feel better	4.3 (0.63)	357 (96.7)	2 (0.5)	10 (2.7)
I would have to change too many habits to follow my medications	4.1 (1.06)	307 (83.2)	1 (0.3)	61 (16.5)
^a^It has been difficult following the medications prescribed for me	4.5 (0.81)	21 (5.7)	3 (0.8)	345 (93.5)
^a^I cannot understand what the doctor told me about my diet (medications)	4.6 (0.72)	13 (3.5)	2 (0.5)	354 (95.9)
^a^Taking my medications interferes with my normal daily activities	4.3 (0.99)	40 (10.8)	2 (0.5)	327 (88.6)

B: Physician–patient relationship	4.4 (0.55)			
I trust my doctor	4.4 (0.67)	357 (96.7)	1 (0.3)	11 (3)
My doctor is dedicated to help me	4.5 (0.57)	363 (98.4)	2 (0.5)	4 (1.1)
I can talk to my doctor	4.4 (0.66)	360 (97.6)	–	9 (2.4)
My doctor has enough time for me	4.3 (0.78)	349 (94.6)	–	20 (5.4)
I feel content with my doctor’s treatment	4.4 (0.71)	357 (96.7)	–	12 (3.3)
I find my doctor easily accessible	4.5 (0.55)	366 (99.2)	–	3 (0.8)
My doctor is dedicated to help me	4.5 (0.55)	367 (99.5)	–	2 (0.5)
My doctor understands me	4.4 (0.56)	366 (99.2)	–	3 (0.8)
My doctor and I agree on the nature of my medical symptoms	4.4 (0.71)	357 (96.7)	1 (0.3)	11 (3)
I benefit from the treatment of my doctor	4.5 (0.55)	365 (98.9)	1 (0.3)	3 (0.8)

	**M (SD)**	**Always ***n*** (%)**	**Do not know, ***n*** (%)**	**Never ***n*** (%)**

C: Health literacy	2.9 (0.59)			
^a^How often do you have problems learning about your medical condition because of difficulty understanding written information?	4.3 (1.45)	68 (18.4)	5 (1.4)	296 (80.2)
How often do you have someone help you read primary health center materials?	3.9 (1.49)	274 (74.3)	2 (0.5)	93 (25.2)
^a^How confident are you filling out medical forms by yourself?	4.5 (1.16)	47 (12.7)	5 (1.4)	317 (85.9)
In general, how easy or hard do you find it to understand medical statistics?	4.6 (0.91)	344 (93.2)	–	25 (6.8)

***^a^**Negatively worded question*.

### Adherence to Medications

Two hundred and fourteen patients (58%) were considered highly adherent (MMAS = 0), 146 (39.5%) were medially adherent (MMAS = 1–2), and nine (2.5%) had low adherence (MMAS ≥ 3). Almost one-third (125; 33.9%) forgot to take their prescribed medications; 39 (10.6%) were careless at times to take the medicine; 28 (7.6%) revealed that they stopped taking the anti-diabetic medications when they felt bad for taking the medications; and 18 (4.9%) reported that they sometimes stopped taking medicines when they felt better. Table 2 presents numbers and percentage of patients who answered “no” by the MMAS-4.

**Table 2:** **Patients’ self-reported adherence to medications by the Morisky Medication Adherence Scale**.

Item	Number of patients answered no (%)
^a^Do you ever forget to take your medicine?	244 (66.1)
^a^Are you careless at times to take your medicine?	330 (89.4)
^a^Sometimes if you feel worse when you take the medicine, do you stop taking it?	341 (92.4)
^a^When you feel better, do you sometimes stop taking your medicine?	351 (95.1)
Adherence to medication (overall)	
**Distribution of scores**	
0	214 (58.0)
1	109 (29.5)
2	37 (10.0)
3	9 (2.5)
4	–

***^a^**Negatively worded question*.

### Factors Influenced High Adherence to Anti-Diabetic Therapies

The number of patients who showed low adherence was very low, and therefore we divided patients into two groups: high adherent group (MMAS score = 0) and intermediate adherent (MMAS score = 1, 2, 3, and 4). At crude analysis using the univariate analysis of independent variables, nine independent variables, including but not limited to age (OR = 1.72, 95% CI: 0.858–3.343), gender (OR = 0.681, 95% CI: 0.447–1.036), duration of DM (OR = 1.046, 95% CI: 0.607–1.803), and health literacy (OR = 1.823, 95% CI: 0.664–5.006) were selected for logistic and multiple linear regression model (*p* ≤ 0.25). Details are presented in Table 3.

**Table 3:** **Factors associated with high adherence to anti-diabetic therapies**.

Variable	Total, *N* = 369	Adherent		Odds ratio with 95% confidence interval	*p*-Value
		High adherent, *N* = 214	Intermediate adherent, *N* = 155		
**Age**					
<45	50 (13.6)	26 (12.1)	24 (15.5)	Reference	0.132
45–60	193 (52.3)	106 (49.5)	87 (56.1)	1.125 (0.603–2.097)	0.712
>60	126 (34.1)	82 (38.3)	44 (28.4)	1.72 (0.885–3.344)	0.110

**Gender**					
Male	163 (44.2)	103 (48.1)	60 (38.7)	Reference	0.073
Female	206 (55.8)	111 (51.9)	95 (61.3)	0.681 (0.447–1.036)	

**Marital status**					
Single	17 (4.6)	10 (4.7)	7 (4.5)	Reference	0.943
Married	352 (95.4)	204 (95.3)	148 (95.5)	0.965 (0.359–2.594)	

**Duration of DM**					
≤5 years	135 (36.6)	72 (33.6)	63 (40.6)	Reference	0.383
6–15 years	149 (40.4)	91 (42.5)	58 (37.4)	0.762 (0.44–1.321)	0.233
≥16 years	85 (23.0)	51 (23.8)	34 (21.9)	1.046 (0.607–1.803)	0.172
Complications					0.674
No	169 (45.8)	100 (46.7)	69 (45.5)	Reference	
Yes	200 (54.2)	114 (53.3)	148 (55.5)	0.915 (0.604–1.386)	

**Hypertension**					0.069
No	319 (86.4)	275 (85.1)	44 (95.7)	Reference	
Yes	50 (13.6)	48 (14.9)	2 (4.3)	3.84 (0.901–16.368)	

**CVD**					
No	341 (92.4)	297 (92)	44 (95.7)	Reference	0.383
Yes	28 (7.6)	26 (8)	2 (4.3)	1.926 (0.442–8.399)	

**Retinopathy**					0.242
No	236 (64)	203 (62.8)	33 (71.7)	Reference	
Yes	133 (36)	120 (37.2)	13 (28.3)	1.501 (0.76–2.963)	

**Neuropathy**					0.309
No	355 (96.2)	312 (96.6)	43 (93.5)	Reference	
Yes	14 (3.8)	11 (3.4)	3 (6.5)	1.501 (0.76–2.963)	

**Nephropathy**					0.054
No	356 (96.5)	314 (97.2)	42 (91.3)	Reference	
Yes	13 (3.5)	9 (2.8)	4 (8.7)	0.301 (0.089–1.02)	

**Type of medication**					
Oral	241 (65.3)	136 (63.6)	105 (67.7)	Reference	0.637
Insulin	85 (23)	53 (24.8)	32 (20.6)	1.279 (0.770–2.124)	0.342
Both	43 (11.7)	25 (11.7)	18 (11.6)	1.072 (0.556–2.069)	0.835

**Previous hospitalization**					
Yes	189 (88.3)	132 (85.2)	321 (87.0)	Reference	0.375
No	25 (11.7)	23 (14.8)	48 (13.0)	1.317 (0.717–2.420)	

**Patient–physician relationship**					0.499
Good	4 (1.1)	3 (1.4)	1 (0.6)	Reference	
Bad	365 (98.9)	211 (98.6)	154 (99.4)	0.457 (0.047–4.433)	

**Health literacy**					
Good	353 (95.7)	146 (94.2)	207 (96.7)	Reference	0.244
Bad	16 (4.3)	9 (5.8)	7 (3.3)	1.823 (0.664–5.006)	

**Health belief model (HBM)**					
No	1 (0.3)	0 (0)	1 (0.6)	Reference	0.999
Yes	368 (99.7)	214 (100)	154 (99.4)	0.000 (0.000 to infinity)	

**HBM: perceived susceptibility**					
No	2 (0.5)	2 (0.9)	0 (0)	Reference	0.999
Yes	367 (99.5)	212 (99.1)	155 (100)	0.000 (0.000 to infinity)	

**HBM: perceived severity**					
No	143 (38.8)	102 (47.7)	41 (26.5)	Reference	0.000
Yes	226 (61.2)	112 (52.3)	114 (73.5)	0.395 (0.253–0.617)	

**HBM: perceived benefit**					
No	12 (3.3)	5 (2.3)	7 (4.5)	Reference	0.252
Yes	357 (96.7)	209 (97.7)	148 (95.5)	1.977 (0.616–6.35)	

### Independent Factors Associated with Adherence to Medications

Findings of logistic regression for binary variables demonstrated factors associated with adherence to anti-diabetic medications were female gender (OR = 1.657, 95% CI: 1.065–2.578) and perceived the severity of DM (OR = 1.510, 95% CI: 0.410–5.560; Table 4). The more the patients with diabetes perceive the severity of DM, the more likely to highly adhere to their regimens. Using the multiple linear regression, two factors predict adherence to anti-diabetic therapies, after adjustment or controlling other factors, were: older age (*t*-test = 1.345) and longer duration of DM (*t*-test = 0.899), but were not statistically significant (Table 5).

**Table 4:** **Logistic regression model for independent variables to predict adherence**.

Variable	*B*	SE	Wald	*p*-Value	Odds ratio with 95% confidence interval
Constant	−0.478	0.825	0.336	0.562	
Gender	0.505	0.226	5.008	0.025	1.657 (1.065–2.578)
Hypertension	0.524	0.342	2.355	0.125	1.689 (0.865–3.300)
Retinopathy	0.156	0.232	0.455	0.500	1.169 (0.742–1.841)
Nephropathy	−0.656	0.600	1.194	0.274	0.519 (0.16–1.683)
Health Literacy	0.690	0.546	1.599	0.206	1.994 (0.684–5.812)
Health belief model (HBM; severity)	−0.990	0.235	17.764	0.000	0.372 (0.234–0.589)
HBM (benefit)	0.466	0.625	0.555	0.456	1.593 (0.468–5.426)

**Table 5:** **Multivariate analysis of factors associated with adherence**.

Predictors	*B*	SE	*t*	*p*-Value	95% confidence interval for *B*	
					Lower	Upper
Constant	0.354	0.138	2.562	0.011	0.082	0.626
Age	0.003	0.003	1.345	0.180	−0.002	0.008
Duration	0.003	0.003	0.899	0.369	−0.004	0.010

## Discussion

In our study, we reported 214 (58%), 146 (39.5%), and nine (2.5%) of primary care patients with type 2 DM, under treatment with either oral medications alone or with insulin, had high, medium, and low adherence, respectively. The information on adherence was based on patients’ recall, and therefore the actual and true prevalence of compliance could be lesser than the presented findings in this study. In addition, patients might have difficulties in remembering their habits and medications taking practices, but this was diminished by asking patients to memorize within a period of 2 weeks only. The reported adherence to this study was suboptimal and lower than previous findings reported from Iran (74.8%) (35), and Nigeria (72.5%) (36). However, Ahmad et al. (37) reported a lower rate of adherence than our finding (47%). The differences in adherence level could be attributed to factors related to the health care settings, and/or socio-economic status, and/or metrics used for adherence assessment. Another possible explanation could be the context of Gaza Strip, in which the treatments are only available for few days from the beginning of each month, and therefore if patients did not get their treatments on time, they have to buy them from own pockets, which overburden the financial and economical responsibility of patients to afford the necessary medications. However, treatments could be available for free in other contexts.

We also found that the level of adherence to medications was associated with patients’ belief about the severity of disease, which reflects the feelings concerning the seriousness of contracting an illness or leaving it untreated. This is enough explained on health psychology model presented in HBM and the Theory of Planned Behaviors (30, 38). The majority of our primary care patients sensitized the severity of DM on their health in future, and therefore 94.6% stated that they are in need for diabetes treatments. Negative beliefs about medications and disease are a potent barrier factors to proper adherence (39, 40). It is necessary to address patients’ belief about the severity of disease, the benefit of treatments, and possible challenging factors, through health education, that contribute to preventing patients from adherence to their therapies. Many studies, from Europe, Africa, and the USA, ensured the importance of having diabetes health education to improve patients’ adherence to their medications (41–43). There is a need to raise patients’ awareness and sensitization about disease and management plan. Possible ways to achieve it could be through the self-management education of diabetes, meeting with a diabetes educator on a regular basis, use of educational materials, establish a community campaign, improve communication between patients and health care providers, and patients’ self-management of diabetes (44).

Females were more likely than males to be adherent to their therapies which is consistent with a research conducted among patients suffering from tuberculosis in Thailand (45). The gender’s effect on adherence rate is contradictory, in which previous studies have shown non-adherence was associated with female (46, 47). A possible explanation for our findings, but remains a source of speculations, is that women are more proactive than men toward following preventive care and seeking medical care to obtain treatments for medical conditions. Moreover, adherence to anti-diabetic medications is challenged with complex regimens especially when comorbidities exist, and so men forget to take their therapies as they are exhausted with work. In addition, a possible higher rate of medications’ side effect could be a reason behind low adherence among men. Also, some studies did not find possible relations between gender and adherence to medications (48, 49).

Old age (≥60 years) was a predictor of good adherence, but the finding was not statistically significant. A recent systematic review conducted by Krueger et al. (50) reported similar findings regarding the relationship between older age (>60 years) and adherence to medications among patients with congestive heart failure. Other studies of patients with heart problems were also in line with our finding (51, 52). Common cause for non-adherence among younger ones could be attributed to new diagnosis (53), limited knowledge of the disease (54), fear of side effect, and burden of regimens (55, 56). Older patients with longer duration of having the disease receive proper support for diabetes management and are more aware of the importance of glycemic control to prevent potential complications and so survive with better quality of life (55). Furthermore, social support given by families in Gaza to be in charge of elderly could be one of the contributing factors to adherence to medications.

Duration of DM was predicted to be an important factor related to adherence to medications but was not statistically significant. The longer the patients have diabetes history, the more likely they will be adherent to the prescribed regimens. This finding is consistent with previous publications (57). A possible explanation is that patients during the early years of disease are not aware of the risk of disease and its serious complications. But when complications start and suffering from disease begins, the attitude toward the disease and related therapies may change leading to a better adherence to medications and providers’ instructions. Furthermore, patients with longer duration of disease are more likely to interact better with their health care providers, understand their treatment plans, and become more aware of their diseases. However, Giemens et al. (58) and Arifulla et al. (59) found a negative relationship between the duration of diabetes and patients’ adherence to medications. Sweileh et al. (60) found no significant relation between the duration of DM disease and adherence to treatments. In view of controversies, we recommend further prospective studies to improve future management and to find out the possible contribution of adherence to medications to patients’ survival.

This study has many limitations: first, the use of self-report method and the simplest and inexpensive method, to evaluate patients’ adherence to anti-diabetic medications. Second, self-report methods usually over-estimate patients’ adherence level. However, Goerge et al. (61) stated that using a valid scale such as the MMAS, the measured adherence level is accurate since the sensitivity and specificity are more than 70%. Third, there was a lack of information about the history of joining educational sessions about diabetes, and the level of education. Fourth, selection bias could happen among primary care patients since those who seek health care are only who care about their health and best. Fifth, reasons for non-adherence were not addressed in this study. The strengths of this study are: first, the randomization applied when selecting PHC and patients; second, gender representativeness in the study sample; and third, the sample size was relatively large and enough to evaluate adherence and factors associated.

### Policy Implications

In the light of study findings, MoH as the stewardship body and responsible for the health of people has to initiate policies and take actions that contribute to the promotion of adherence to medications among type 2 DM. Among these policies are: improving the provider–patient relationship and building a trust relation; making the treatments accessible at the time of use; enhancing the self-management and self-monitoring of blood glucose; and providing patients with a degree of autonomy motivation to ensure optimum glycemic control. Moreover, MoH should establish a diabetes education service to raise patients’ awareness of DM and enhance health belief perception toward the benefit of treatments, severity, and susceptibility of non-adherence in the occurrence of complications.

## Conclusion

This study showed that patients’ adherence to anti-diabetic medication was suboptimal and variations were found between high adherent and intermediate adherent with regard to, but not limited to, age, gender, duration of DM, health literacy, health belief, and the absence of co-morbidities. The longer duration of having the disease, old age, female gender, and patient’s self-perception and belief about the severity of DM are factors associated with adherence to medications. Enhancing patients’ knowledge and recognition with diabetes, through diabetes educator, is necessary to improve self-management of DM and increase the rate of adherence. Increasing interactions and have a partner relationship with patients are keys to improve patients’ adherence to medications.

## Ethics Statement

This study received the ethical approval from Palestinian Ethical Research Committee (No: PHRC/HC/118/16 on 06/Jun/2016). This is an independent regional body that comprises members of government and external professionals and researchers. A permission was also obtained from Ministry of Health to visit the selected primary health care centers and conduct the study. Patients were informed about their right to withdraw from the study at anytime without any harmful consequences. Moreover, we verbally stressed on the voluntary participation in the study, giving the right to withdraw at any time without any consequences. The study was conducted over a period of 3 months starting from October 2016.

## Author Contributions

AE, MR, and HS developed the conception and design of this study; AA analyzed and interpreted the analyzed data; AE drafted the manuscript; AE, MR, AA, and HS revised the manuscript, provided final approval of the manuscript, and approved this manuscript.

## Conflict of Interest Statement

The authors declare that there is no conflict of interests regarding the publication of this paper.
